# Metal-free magnetism, spin-dependent Seebeck effect, and spin-Seebeck diode effect in armchair graphene nanoribbons

**DOI:** 10.1038/s41598-018-19632-3

**Published:** 2018-01-17

**Authors:** Xiao-Qin Tang, Xue-Mei Ye, Xing-Yi Tan, Da-Hua Ren

**Affiliations:** School of Science, Hubei University for Nationalities, Enshi, 445000 People’s Republic of China

## Abstract

Metal-free magnetism and spin caloritronics are at the forefront of condensed-matter physics. Here, the electronic structures and thermal spin-dependent transport properties of armchair graphene nanoribbons (*N*-AGNRs), where *N* is the ribbon width (*N* = 5–23), are systematically studied. The results show that the indirect band gaps exhibit not only oscillatory behavior but also periodic characteristics with *E*_*3p*_ > *E*_*3p*+*1*_ > *E*_*3p*+*2*_ (*E*_*3p*_, *E*_*3p*+*1*_ and *E*_*3p*+*2*_ are the band gaps energy) for a certain integer *p*, with increasing AGNR width. The magnetic ground states are ferromagnetic (FM) with a Curie temperatures (*T*_*C*_) above room temperature. Furthermore, the spin-up and spin-down currents with opposite directions, generated by a temperature gradient, are almost symmetrical, indicating the appearance of the perfect spin-dependent Seebeck effect (SDSE). Moreover, thermally driven spin currents through the nanodevices induced the spin-Seebeck diode (SSD) effect. Our calculation results indicated that AGNRs can be applied in thermal spin nanodevices.

## Introduction

Spin caloritronics, combining spintronics and thermoelectronics, plays an extremely important role in the development of fundamental science and novel low-power-consumption technologies^1–7^. In this field, Uchida *et al*. made the pioneering discovery of the spin Seebeck effect (SSE), in which a spin current and an associated spin voltage are induced only by a temperature gradient^8^. To use SSE in practical device applications^9–11^, the spin-Seebeck diode (SSD) effect^12–14^, which allows the thermal-induced spin currents to flow in only one direction and be rectified, is required. In recent years, there have been many reports concerning these effects^15–18^. Zeng *et al*. demonstrated that opposite spin currents can be generated in magnetized zigzag graphene nanoribbons (M-ZGNRs) by a temperature difference between the source and the drain^19^. Ni *et al*. observed the SSE and the thermal colossal magnetoresistance effect (CMR) in zigzag graphene nanoribbons (ZGNRs)^20^. Until now, almost all the related studies concern ZGNRs^11,21–26^, and there are a few reports focusing on AGNRs due to their nonmagnetic properties. Simbeck *et al*. found that the electronic and magnetic behavior of oxygen-functionalized AGNRs determines their geometry and that planar systems have spin-polarized ground states^27^. Soriano *et al*. introduced magnetism in AGNRs by adsorbing H atoms to the C atoms at the ribbon center^28^. Nguyen *et al*. investigated the spin-dependent transport in AGNRs controlled by an FM gate by connecting AGNRs to FM metal leads for spin transmission^29^. In addition, Zhang *et al*. recently found that AGNRs with edge hydrogenation behave as bipolar magnetic semiconductor (BMS), opening a new avenue for spintronic devices^30^. On the other hand, AGNRs are more stable than ZGNRs, according to Okada’s calculation results^31^. Thus, it is worthwhile to investigate the practical application of AGNRs to spin caloritronics devices. In this paper, using ab initio calculations combined with the nonequilibrium Green’s function approach^32,33^, the thermal spin-dependent transport characteristic of AGNRs were studied. The numerical calculations demonstrated that apart from the perfect SSE, the SSD effect can also be obtained. Our results indicate that AGNRs are promising for application in spin caloritronic devices.

## Results

**Figure 1:**
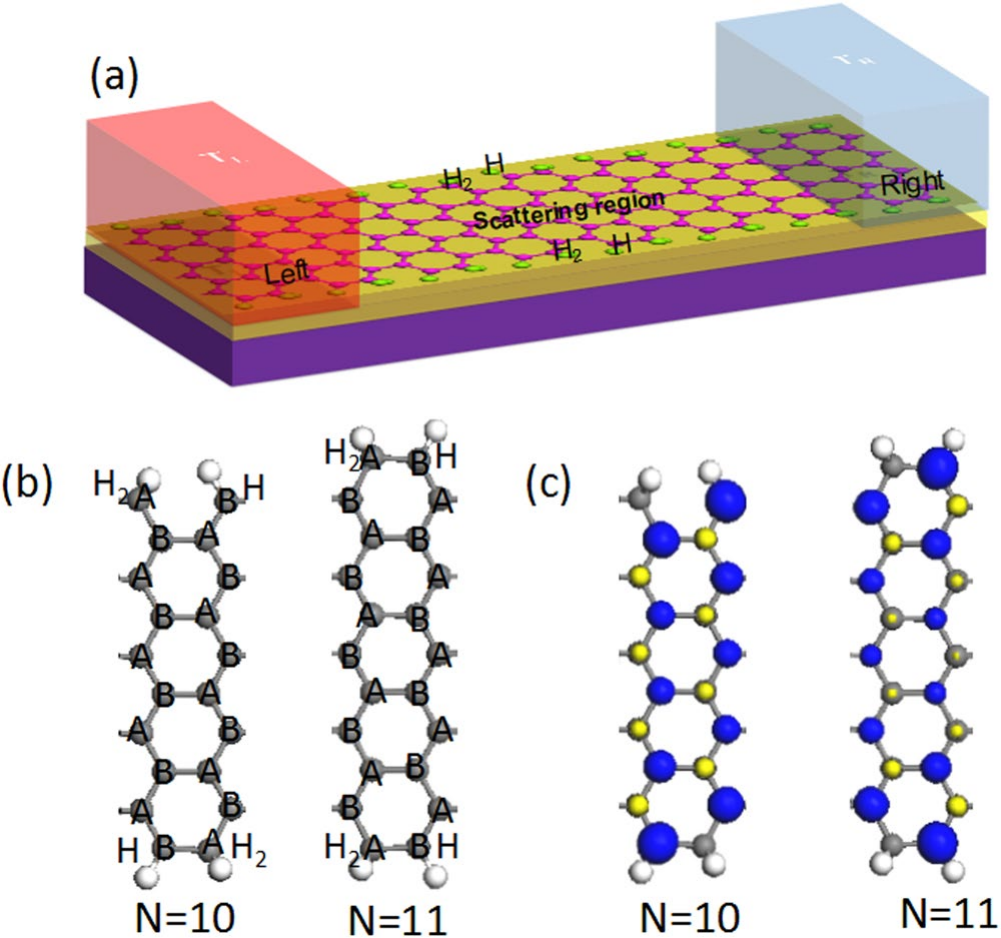
(**a**) Schematic illustration of the thermal spin device based on *N*-AGNRs (*N* = 9 as an example). (**b**) Optimized structure of the *N*-AGNRs (*N* = 10, 11 as an example), where the black and white balls indicate carbon and hydrogen atoms, respectively. (**c**) Isosurface of the spin density of the ferromagnetic state with an isovalue of 0.05 eV/Å^3^ (*N* = 10, 11 as an example), where blue and yellow indicate positive and negative values, respectively.

As we well know, two kinds of sublattices appear alternately in graphene: one with a type A carbon atom and one with a type B carbon atom at the edge. We passivated all type A carbon atoms at both edges by two H atoms, and the type B carbon atoms by one H atom, as shown in Fig. 1(b) (*N* = 10, 11 as examples). These special AGNRs can be fabricated experimentally because the progress of hydrogenation can be controlled by the chemical potential of hydrogen via the temperature and pressure of the H_2_ gas^34–36^. Then, we built two-probe spin caloritronics devices based on the *N*-AGNRs (*N* = 9 as an example), as shown in Fig. 1(a). The left and right electrodes are semi-infinite and are connected by a finite length of graphene nanoribbons in the middle. The temperature applied at the source is denoted *T*_*L*_, while that applied at the drain is denoted *T*_*R*_; therefore, the temperature gradient is *ΔT* = *T*_*L*_*-T*_*R*_.

**Figure 2:**
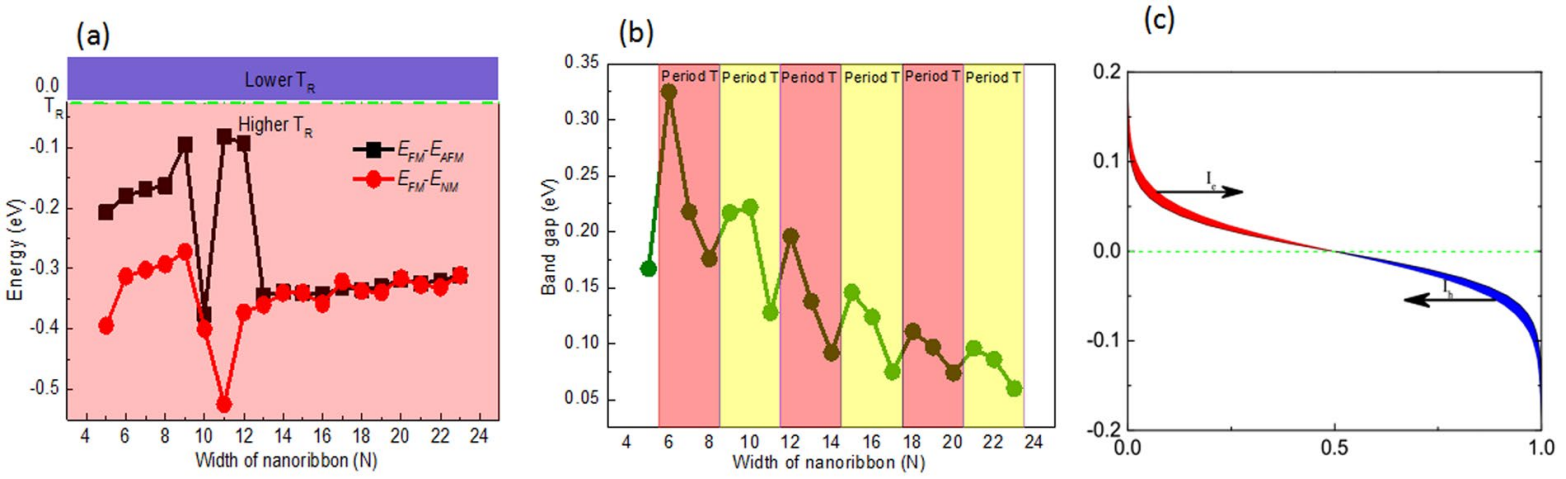
(**a**) The energies *E*_*FM*_-*E*_*AFM*_ and *E*_*FM*_-*E*_*NM*_ of the *N*-AGNRs (*N* = 5–23), where red and black indicate *E*_*FM*_-*E*_*NM*_ and *E*_*FM*_-*E*_*NM*_, respectively. (**b**) Variation in the band gap energy as a function of the width of the *N*-AGNRs (*N* = 5–23). (**c**) Fermi distributions of the left (**a**) and right (**c**) leads, where *T*_*L*_ = 300 K and *T*_*R*_ = 298 K.

To study the magnetic ground state of the AGNRs, three different magnetic states, ferromagnetic state (FM), antiferromagnetic state (AFM), and nonmagnetic state (NM), were calculated. We use the labels *E*_*FM*_, *E*_*AFM*_ and *E*_*NM*_ to denote the total energies per unit cell for the FM, AFM and NM configurations, respectively. Then, the energies *E*_*FM*_-*E*_*AFM*_ and *E*_*FM*_-*E*_*NM*_ were calculated and found to be negative, as shown in Fig. 2(a). In other words, the FM are the ground states of AGNRs. Using the mean field theory^37^, the Curie temperatures (*T*_*C*_) were estimated. The relationship between *T*_*C*_ and *E*_*AFM*_-*E*_*FM*_ is γk_B_*T*_*C*_/2 =* E*_*AFM*_-*E*_*FM*_, where γ and k_B_ represent the dimension of the system (we set γ to 1 because the graphene nanoribbons are one-dimensional materials) and the Boltzmann constant, respectively. From Fig. 2(a), we can see that the *T*_*C*_ of all AGNRs are above room temperature (26 meV). The distribution of the spin density was further studied; the type A and type B carbon atoms in the nanoribbons have opposite spins, and the lower edge carbon atoms have larger magnetic moments, as shown in Fig. 1(c) (*N* = 10, 11 as examples). The total spin magnetic moment is 2 *μ*_*B*_ for each ribbon. This result can be easily understood by Lieb’s theorem^38^, in which the magnetic moment is determined by the sublattice difference of a bipartite lattice.

**Figure 3:**
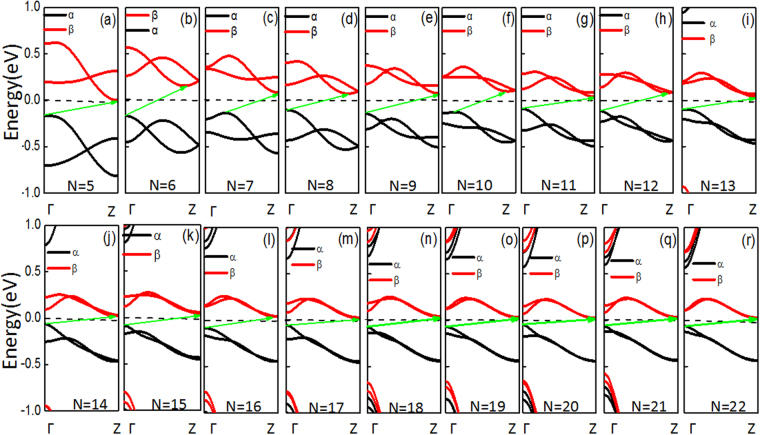
Spin-dependent band structures of the *N*-AGNRs (*N* = 5–22).

To further trace the origins of the physical mechanisms, we investigated the band structures of the *N*-AGNRs (*N* = 5~22), as shown in Fig. 3. The bottom conduction bands are related to the β-spin states, which are above the Fermi level (*E*_*F*_), and the α-spin states below *E*_*F*_ serve as the top valence band. The valence and conduction bands possess opposite spin polarization as they approach *E*_*F*_. Moreover, the spin-dependent bands of all the AGNRs have finite gaps around *E*_*F*_. These characteristics indicate that these AGNRs are BMS^30,39,40^. In addition, it is noted that BMS appeared irrespective of the ribbon width. Furthermore, it is clear that all the AGNRs are indirect semiconductors. Form Fig. 3, one can find that the positions of the bottom conduction bands gradually increase and eventually approach the Z point when *N* is even. However, when *N* is odd, the positions of the bottom conduction band are maintained at the Z point. In other words, the positions of the bottom conduction bands fluctuate between the Z point and a certain value when 5 ≤ *N* ≤ 16 but are located at the Z point for 17 ≤ *N* ≤ 22. The positions of top valence bands have three cycles when 5 ≤ *N* ≤ 10. When *N* = 5, 6, 8 and 9, they are located at the Γ point, while for* N* = 7 or 10, they are located at a certain value. When *N* ≥ 11, the positions of the top valence bands are located at the Γ point. For AGNRs, the variations in the band gap with ribbon width (*N* = 5~23) are summarized in Fig. 2(b). It is obvious that the band gaps of the AGNRs show an oscillatory behavior when *N* = 6~23, meaning that the band gaps exhibit periodic characteristics with *E*_*3p*_ > *E*_*3p*+*1*_ > *E*_*3p*__+*2*_ for a certain integer p, except when p is equal to 3. For example, the band gap of 6-AGNRs is 0.325 eV, and those of 7-AGNRs and 8-AGNRs are 0.218 eV and 0.176 eV, respectively. Similar oscillatory behavior was also observed in H-passivated AGNRs^41^ and buckled ASiNRs^42,43^.

**Figure 4:**
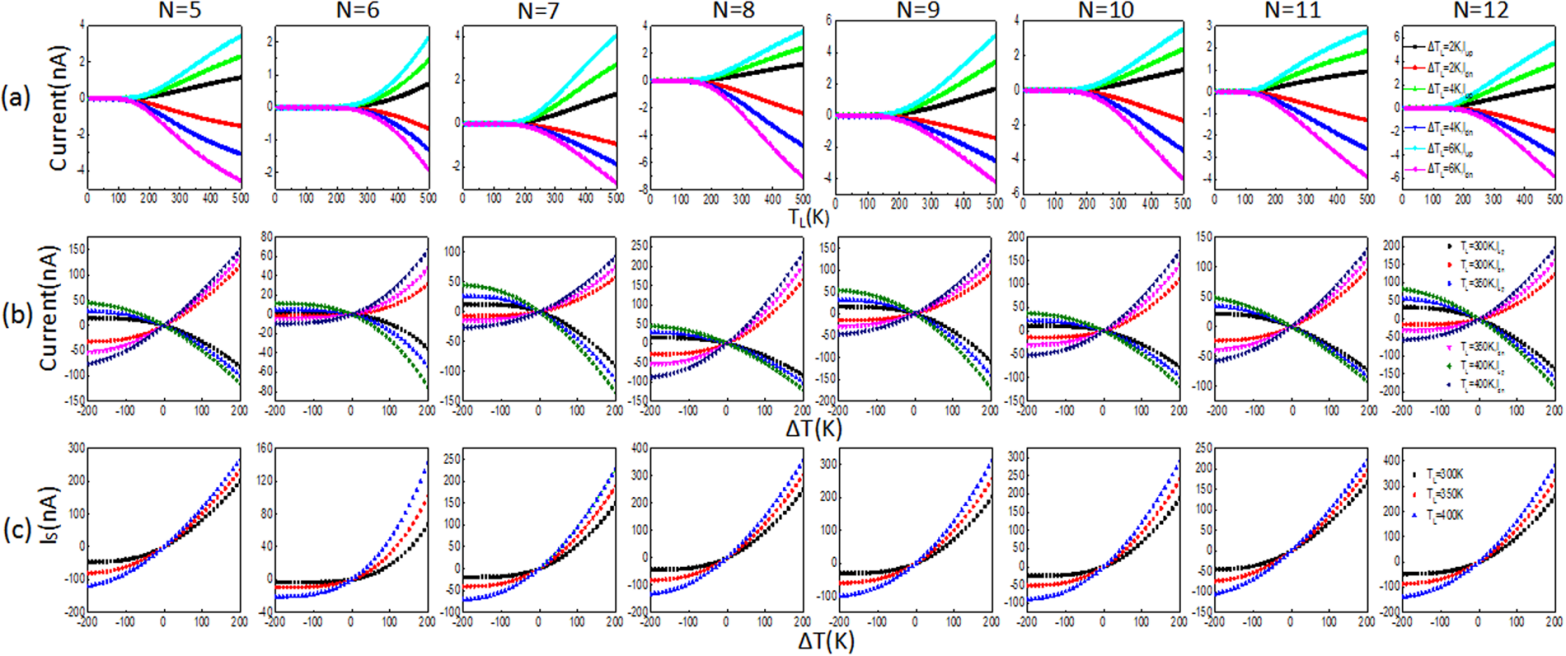
(**a**) Spin-up currents (*I*_*up*_) and spin-down currents (*I*_*dn*_) as a function of *T*_*L*_ for the *N*-AGNRs (*N* = 5–12) at selected temperature gradients. (**b**) *I*_*up*_ and *I*_*dn*_ versus *ΔT* for selected values of *T*_*R*_. (**c**) Net spin currents *I*_*s*_ (=*I*_*up*_−*I*_*dn*_) as a function of the temperature gradient *ΔT*.

Figure 4(a) shows the spin-dependent currents through the *N*-AGNR (*N* = 5–12) devices versus *T*_*L*_, with *ΔT* set to 2, 4 and 6 K. Apparently, there are no spin-up currents (*I*_*up*_) or spin-down currents (*I*_*dn*_) when *T*_*L*_ < 200 K for the three values of *ΔT*, which suggests that no thermal-induced spin-dependent currents are generated in this range of *T*_*L*_, no matter how large the temperature difference (*ΔT*). In other words, there is a threshold temperature *T*_*th*_ at approximately 200 K for both *I*_*up*_ and *I*_*dn*_. When *T*_*L*_ > *T*_*th*_, both *I*_*up*_ and *I*_*dn*_ increased sharply with increasing *T*_*L*_. However, they flow in the opposite directions, i.e., *I*_*up*_ is positive and *I*_*dn*_ is negative. There is no doubt that this is caused by the SSE^20^. Furthermore, the higher the *ΔT*, the larger the spin-dependent currents. The spin-dependent currents versus *ΔT* curves are plotted in Fig. 4 (b), with *T*_*L*_ set to 300, 350 and 400 K. The curves clearly indicate that the spin-dependent currents are symmetric about the zero-current axis and robust over a large range of temperature gradients. In addition, in all the AGNRs, when *ΔT* < 0 K the spin-dependent currents are miniscule, otherwise the spin-dependent currents increased quickly with the increasing *ΔT*. Therefore, the SSE can also be confirmed by the spin-dependent currents versus *ΔT* curves. To shed more light on the spin-dependent currents, the total spin current *I*_*s*_ ( = *I*_*up*_*-I*_*dn*_) versus *ΔT* is plotted in Fig. 4(c). One can find that when *ΔT* < 0 K, *I*_*s*_ approaches zero. When *ΔT* > 0 K, *I*_*s*_ continues to increase and is much larger than that when the temperature is below zero. These characteristics indicate that the spread of *I*_*s*_ is allowed to flow in only one direction, which indicates the SSD effect.

**Figure 5:**
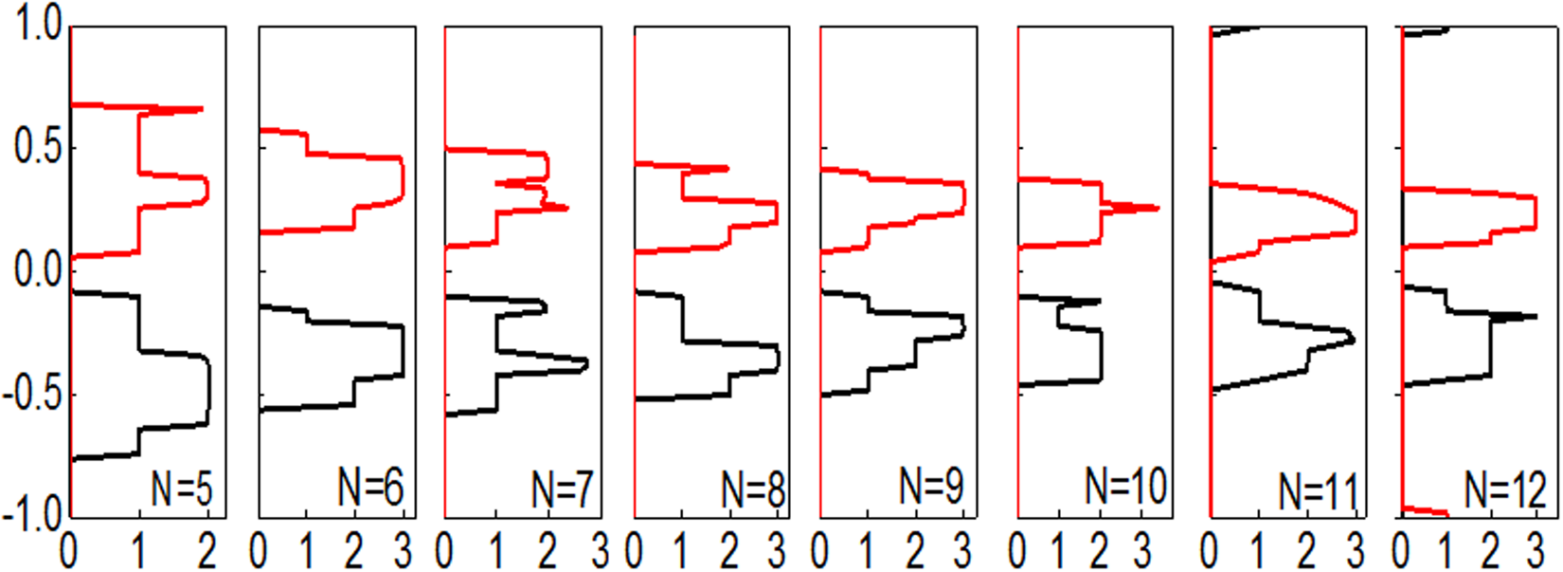
Spin dependent transmission spectra of the *N*-AGNRs (*N* = 5–12), where the solid gray line indicates spin-up and the solid red line indicates spin-down.

To illustrate the underlying mechanism of these phenomena, the carrier distributions were considered. As the two lead regions and the scattering region are composed of the same material and possess the same band structures, the difference in carrier concentrations between the left and right leads are determined only by the Fermi distribution, which is intimately related to the temperature of the two leads. To give a direct illustration, we plot the Fermi distributions of the left and right leads in Fig. 2(c). It is clear that carriers with higher energy than the Fermi energy flow from left to right due to the difference in the Fermi distribution, generating an electron current (*I*_*e*_). Conversely, carriers with energy lower than the Fermi energy flow in the opposite direction, resulting in a hole current (*I*_*h*_). Based on the Landauer–Büttiker formula^44^, when the transmission for each spin is independent of energy, *I*_*e*_ and *I*_*h*_ will be equal, and SSE will not appear. However, for *N*-AGNRs, the spin-dependent transmission depends on the energy; as shown in Fig. 5, the spin-up subbands are below the *E*_*F*_, spin-down subbands are above the *E*_*F*_. This broke the electron-hole symmetry, leading to nonzero spin currents, and the SSE appeared in these devices. Furthermore, the spin-resolved transmission spectra show a spin band gap, leading to transmission channels open when *T*_*L*_ increases to a critical temperature (*T*_*c*_). Owing to the existence of a spin-splitting band gap, the transmission channels open only when *T*_*L*_ increases to a critical value *T*_*c*_. When *T*_*L*_ < *T*_*c*_, the populations of electrons and holes in the source are low, and the transmission channels are essentially closed. Due to this mechanism, if we set *T*_*L*_ to a chosen value, the transmission channels open and the spin-dependent currents are generated only when *T*_*R*_ decreases to a critical value; otherwise, the spin-dependent currents approach zero due to the existence of a spin-splitting band gap. As a result, the SSD effect of the spin-dependent currents emerges.

## Discussion

In summary, we investigated the electronic structures and thermal spin-dependent transport properties of a series of *N*-AGNRs by first-principles calculations combined with the nonequilibrium Green’s function. First, all the *N*-AGNRs behave as indirect semiconductors and have FM ground states. Furthermore, *I*_*up*_ and *I*_*dn*_ were generated only by a temperature gradient and were found to have opposite directions, indicating the appearance of the perfect SSE. Moreover, an SSD effect can also be found in these nanodevices. Apart from the above, it is found that the effects of substrate (hexagonal boron nitride) are not important. In general, these findings strongly suggest that *N*-AGNRs are promising materials for thermal spin nanodevices.

## Methods

In the calculations, geometry optimization and electronic structure calculations were performed with the double numerical plus polarization (DNP) basis set implemented in the SIESTA code^45^. The positions of the atoms were relaxed until the maximum force on each atom was no more than 0.05 eV Å^-1^. Then, we calculated the transmittances using the TRANSAMPA code^46,47^. The core electrons were described by norm-conserving pseudo- potentials and the local density approximation (LDA)^48^. A double-zeta-polarized (DZP) basis set was used, the cut off energy was 150 Ry, and a Monkhorst–Pack 1 × 1 × 100 k-mesh was chosen. In the Landauer–Büttiker formalism, the spin-dependent current through the system is given by the equation^46^

1 $${I}^{\uparrow (\downarrow )}=\frac{e}{h}{\int }_{-\infty }^{\infty }\{{T}^{\uparrow (\downarrow )}(E)[{f}_{L}(E,{T}_{L})-{f}_{R}(E,{T}_{R})]\}dE$$

where e is the electron charge, h is Plank’s constant, *f*_*L*(*R*)_(*E*, *T*_*L*(*R*)_) is the equilibrium Fermi–Dirac distribution for the left (right) lead, *T*_*L*(*R*)_ is the temperature of the left (right) contract, and *T* ^↑(↓)^(*E*) is the spin-resolved transmittance function, which can be defined as

2 $${T}^{\uparrow (\downarrow )}(E)=Tr{[{{\rm{\Gamma }}}_{L}{G}^{R}{{\rm{\Gamma }}}_{R}{G}^{A}]}^{\uparrow (\downarrow )}$$

where G^R(A)^ is the retarded (advanced) Green’s functions of the central region and _Γ*L*(*R*)_ is the coupling matrix of the left (right) contact.
